# Study on the application effect of traditional Chinese medicine syndrome differentiation nursing in the rehabilitation of language function in patients with stroke

**DOI:** 10.1097/MD.0000000000041344

**Published:** 2025-02-21

**Authors:** JingTiao Lu, Jing Mi, Jia Zhou

**Affiliations:** a Department of Neurorehabilitation, Kunshan Rehabilitation Hospital, Kunshan, Jiangsu, China; b Nursing Department, Xiangcheng Hospital of Traditional Chinese Medicine, Suzhou, Jiangsu, China; c Department of Acupuncture and Rehabilitation, Xiangcheng Hospital of Traditional Chinese Medicine.

**Keywords:** BDAE score, clinical efficacy evaluation, language function rehabilitation, stroke, traditional Chinese medicine syndrome differentiation nursing

## Abstract

This study aimed to investigate the efficacy of traditional Chinese medicine (TCM) syndrome differentiation nursing in the rehabilitation of language function in stroke patients. This study compares the effects of TCM syndrome differentiation nursing and conventional rehabilitation nursing by assessing changes in language function using the Boston Diagnostic Aphasia Examination (BDAE) scores and clinical efficacy evaluation. This study is a retrospective study in which we reviewed cases of poststroke rehabilitation from 2020 to 2023. A total of 100 stroke patients were enrolled and divided into 2 groups: the TCM syndrome differentiation nursing group (n = 50) and the conventional rehabilitation nursing group (n = 50). TCM syndrome differentiation nursing was tailored based on individual patients’ TCM diagnoses (e.g., Qi deficiency, blood stasis, or phlegm-dampness) and involved a combination of targeted nursing care, acupuncture, and herbal remedies. The conventional rehabilitation nursing group received standard poststroke rehabilitation therapies, including speech therapy and physical exercises. Both groups were assessed using BDAE scores and clinical efficacy evaluations at baseline and after 12 weeks of intervention. The TCM syndrome differentiation nursing group showed significantly greater improvements in BDAE scores compared with the conventional rehabilitation group (mean increase: 15.2 vs 8.6, *P* < .05). In addition, 72% of patients in the TCM group were classified as having “marked improvement” in clinical efficacy, compared with 48% in the conventional rehabilitation group (*P* < .05). The TCM group also reported fewer complications such as fatigue and stress compared with the conventional rehabilitation group (*P* < .05). TCM syndrome differentiation nursing significantly enhances language function recovery in stroke patients and serves as a valuable supplement to conventional rehabilitation methods. This study provides novel evidence supporting the integration of TCM-based interventions in stroke rehabilitation, offering a complementary approach to existing therapeutic strategies. Further research is needed to explore the long-term effects and underlying mechanisms of TCM syndrome differentiation nursing.

## 1. Introduction

Stroke is a common neurological disorder, with its incidence and disability rates showing an increasing trend globally, significantly affecting patients’ quality of life and socioeconomic development.^[1-3]^ In the rehabilitation treatment of stroke, the recovery of language function is an important yet complex task. Although modern medicine has made certain advancements in the treatment of stroke, research on the application of traditional Chinese medicine (TCM) syndrome differentiation nursing in the rehabilitation of language function in stroke patients is relatively scarce. Therefore, this study aimed to explore the application effect of TCM syndrome differentiation nursing in the rehabilitation of language function in stroke patients, providing new insights and methods for the rehabilitation treatment of stroke patients.^[4-6]^

As an important component of TCM treatment, TCM syndrome differentiation nursing emphasizes individualized treatment principles tailored to each person and each situation,^[7]^ focusing on the comprehensive analysis of the patient’s overall health status and syndrome differentiation treatment. In the rehabilitation process of stroke patients, TCM syndrome differentiation nursing can not only regulate the patient’s qi, blood, yin, and yang to promote the recovery of bodily functions but also further enhance the rehabilitation effect through individualized nursing interventions targeting different types of patients.^[4,7,8]^

This study will systematically investigate and analyze the application effect of TCM syndrome differentiation nursing in the rehabilitation of language function in stroke patients from the aspects of research trial methods, technical routes, and processes. First, we will clarify the recruitment criteria and inclusion criteria of the research subjects to ensure the rigor and credibility of the study. Second, we will introduce the specific contents of conventional rehabilitation nursing and TCM syndrome differentiation nursing, as well as the setting of observation indicators and efficacy evaluation methods. Finally, statistical analysis methods will be used to objectively evaluate and analyze the research results, validating the actual application effect of TCM syndrome differentiation nursing in the rehabilitation of language function in stroke patients. Through the implementation of this study, we hope to provide scientifically based TCM syndrome differentiation nursing schemes for clinical practice, offering more effective methods and strategies for the rehabilitation treatment of stroke patients, thereby further improving the rehabilitation rate and quality of life of patients.

## 2. Methods

### 2.1. Study subjects

Patients recruited for this study must meet the following criteria. First, in terms of diagnosis, they must undergo magnetic resonance imaging, head computed tomography scans, and other relevant examinations, satisfying the diagnostic criteria for stroke outlined in the “Clinical Guidelines for Diagnosis and Treatment of Cerebrovascular Diseases.” Second, the inclusion criteria include patients who meet the diagnostic standards for acute ischemic stroke according to the “Guidelines for the Diagnosis and Treatment of Acute Ischemic Stroke in China 2014” and criteria for syndrome differentiation in TCM; confirmation by computed tomography/magnetic resonance imaging at our hospital; age between 18 and 80 years; clear consciousness and basically stable vital signs; ability to cooperate with treatment and adhere to follow-up visits; and voluntary signing of informed consent forms by both patients and their families. However, patients with intellectual disabilities, malignant brain tumors, mental disorders, severe heart, liver, or kidney damage, and elderly individuals who live alone or lack caregivers will be excluded from this study.

Retrospectively, the subjects will be divided into 2 groups based on their different treatment approaches: a control group and a study group, with 50 cases in each group. This grouping method will help us explore the impact of different treatment methods on stroke patients more precisely and provide a more reliable basis for clinical practice. The research project has been reviewed and approved by the Ethics Committee of Kunshan Rehabilitation Hospital, and clinical research registration has been completed. All research subjects or their guardians have been informed of the purpose and significance of the study, voluntarily participated in the study, and signed informed consent forms.

### 2.2. Conventional rehabilitation nursing program

The following is a detailed description of the routine rehabilitation nursing program, encompassing muscle function exercises, dietary care, psychological care, and medication care.

Muscle function exercises play a vital role in rehabilitation nursing, aiming to enhance patients’ muscle strength, improve muscle coordination and flexibility, and promote the recovery of physical functions. A comprehensive assessment of the patient’s muscle function is conducted first to understand their muscle strength, coordination, and flexibility. Based on this assessment, a personalized exercise plan is developed, specifying the specific exercises, frequency, intensity, and duration to meet the patient’s rehabilitation needs. Under the guidance of rehabilitation professionals, patients perform these exercises, ensuring that the movements are standardized and safe to avoid excessive strain and potential injury. Regular monitoring of the patient’s muscle function recovery is conducted, and the exercise plan is adjusted accordingly to ensure optimal rehabilitation outcomes.

Dietary care is also crucial in the rehabilitation process. A balanced diet helps patients replenish nutrients, strengthen their bodies, and promote recovery. An individualized diet plan is tailored to the patient’s physical condition, rehabilitation needs, and nutritional requirements, incorporating appropriate calories, proteins, fats, vitamins, and minerals. Patients and their families receive dietary guidance on selecting suitable foods, creating balanced meals, and avoiding unhealthy eating habits. Regular monitoring of the patient’s nutritional status is conducted, and the diet plan is adjusted based on the progress of rehabilitation and changes in nutritional needs.

Psychological care is an integral part of rehabilitation nursing, aimed at helping patients adjust their mindset, reduce psychological pressure, and enhance rehabilitation outcomes. Through psychological assessments, we understand the patient’s mental state, emotional changes, and psychological needs. Psychological counseling and comfort are provided to address patients’ psychological issues, fostering a positive and optimistic attitude and enhancing their confidence in recovery. We also provide psychological support, including listening to the patient’s concerns, addressing their doubts, and encouraging their active participation in rehabilitation activities. Education on psychological health is also provided to patients and their families, teaching them how to cope with psychological challenges and maintain emotional balance during the rehabilitation process.

Medication care ensures the safe and rational use of medications for patients, crucial for promoting recovery. Patients and their families are informed about the medications, including their names, usage, dosage, and potential side effects, to ensure proper medication use. Based on doctors’ prescriptions and the patient’s physical condition, medication guidance is provided, including when to take the medication, the order of administration, and any special precautions. Close observation of the patient’s response to medication is conducted, and prompt action is taken in case of adverse reactions or allergic reactions, with immediate reporting to the doctor. In addition, we assist patients in managing their medications, ensuring safe and effective storage to prevent expiration or deterioration.

A total of 100 stroke patients were included in this study and divided into a TCM syndrome differentiation nursing group (50 patients) and a routine rehabilitation nursing group (50 patients). The sample size was calculated based on the expected medium effect size (Cohen *d* = 0.5), taking into account a statistical power of 80% and a significance level of 0.05. Sample size estimates using the G*Power software, based on the effect size in previous similar studies, showed that each group of 50 people was able to detect significant differences in language function recovery at a set level of significance. In addition, given the nature of the study design, the sample size was set to 100 participants to ensure sufficient statistical power to reliably assess the impact of TCM syndrome differentiation care on speech function recovery.

### 2.3. TCM syndrome differentiation rehabilitation nursing program

1. Conduct TCM syndrome differentiation analysis on patients and divide them into 3 types based on clinical manifestations and etiology: Qi deficiency and blood stasis type, liver and kidney deficiency type, wind-phlegm obstruction type. Provide targeted nursing interventions for different types of patients during TCM syndrome differentiation nursing. Therapy sessions were conducted for 45 minutes, 5 times per week, for a duration of 12 weeks. The intensity and approach were adjusted based on the patient’s baseline language function as assessed by the Boston Diagnostic Aphasia Examination (BDAE).

- Qi deficiency and blood stasis type: Encourage patients to read books and newspapers aloud to strengthen the muscles around the mouth, instruct patients to wash their face and feet with hot water daily to stimulate peripheral blood circulation, promote tendon relaxation, and invigorate Qi and activate blood circulation.
- Liver and kidney deficiency type: Ensure patients get sufficient rest and maintain a quiet ward environment; encourage exercises such as chewing gum and eating melon seeds to exercise the muscles around the mouth; ensure balanced nutrition in meals with supplements such as Chinese wolfberry, Chinese yam, and *Angelica sinensis* soup; provide foot massages before sleep.
- Wind-phlegm obstruction type: Strengthen emotional intervention for patients and guide family members to communicate more with patients to maintain a relatively pleasant mood; advocate a light diet, avoiding spicy and greasy foods, as well as phlegm-producing foods; encourage phlegm expectoration.

2. Implement characteristic TCM syndrome differentiation rehabilitation nursing programs for patients based on conventional nursing methods, including syndrome differentiation dietary therapy, emotional adjustment with 5 tones, meridian tapping, and acupressure massage.

- Syndrome differentiation dietary therapy: Tailor dietary plans based on individual patient conditions, syndrome types, constitution types, seasonal climates, and regional variations, aiming to fundamentally improve patients’ physical condition and balance Yin and Yang, thereby improving clinical symptoms and enhancing patients’ quality of life.
- Emotional adjustment with 5 tones: Music therapy, based on the principle that “hundreds of diseases arise from Qi and are stopped by music” in the “Yellow Emperor’s Inner Canon,” plays 5 tones (Gong, Shang, Jiao, Zhi, Yu) in a loop every day to divert patients’ attention, relieve negative emotions, regulate the Qi mechanism of the 5 Zang organs, and significantly reduce the recurrence rate of stroke.
- Meridian tapping: Tapping the head promotes blood circulation, enhances memory and cognitive function, invigorates Yang Qi, and improves the functions of the Zang-fu organs; tapping along the body’s surface and limbs helps smooth Qi and blood circulation, improve the functions of the Zang-fu organs, and enhance motor and sensory functions.
- Acupressure massage: Applying herbal patches to specific acupoints such as Tianzhu, Lianquan, Renying, Yamen, Jia Che, and Di Cang helps improve facial symmetry and speech difficulties; acupoints such as Neiguan, Quchi, Jianyu, Jiquan, and Hegu promote nerve function recovery, improving upper limb movement and sensory functions; acupoints like Zusanli, Sanyinjiao, Weizhong, Yanglingquan, and Huan Tiao facilitate lower limb function recovery, improving lower limb movement and sensory functions; acupoints such as Taiyang and Taixi nourish the liver and kidneys and extinguish wind to alleviate symptoms of liver wind, while Fenglong, Qihai, and Xuehai tonify Qi and invigorate blood to resolve phlegm and stasis.

### 2.4. Observation indicators

1. Use the “BDAE” to grade patients’ language function: Level 0: Unable to communicate; Level I: Able to say a few words; Level II: Able to speak simple sentences but with grammatical errors; Level III: Able to communicate in some situations but often encounters difficulties; Level IV: Communication is mostly normal but may not be fluent at times; Level V: Mild impairment, not easily noticeable by the interlocutor.
2. Evaluation of therapeutic efficacy: Clinical cure: BDAE level V; significant improvement: increase in BDAE level by 2 grades; effective: increase in BDAE level by 1 grade; ineffective: no improvement after intervention.
3. A comparison of language ability scores before and after nursing between the 2 groups was conducted. The scores were evaluated using the Chinese aphasia examination, with higher scores indicating better language ability in patients.
4. A comparison of depression and anxiety scores before and after nursing was made between the 2 groups. Anxiety scores were assessed using the Self-rating Anxiety Scale (SAS), while depression scores were evaluated using the Self-rating Depression Scale (SDS).
5. A comparison of quality of life scores before and after nursing was conducted between the 2 groups, utilizing the quality of life rating scale for evaluation. Higher scores indicated poorer quality of life in patients.

### 2.5. Statistical analysis methods

SPSS 30.0 statistical software is used for data analysis. The Shapiro–Wilk test was used to test the normality of the data. For normally distributed data, parametric tests (*T* tests) are used to compare group differences because these tests assume that the data is normally distributed. If the data is found to be nonnormal distribution, nonparametric tests (such as Mann–Whitney *U* test and Wilcoxon sign-rank test) are used as appropriate.

In this article, the data conform to normal distribution. For quantitative data, the *t* test will be used; for ranked data, the rank sum test will be used, with *P* < .05 considered statistically significant.

## 3. Results

### 3.1. Baseline data

There were no statistically significant differences in gender, age, or BDAE grading between the 2 groups (*P* > .05), indicating comparability.

### 3.2. A comparison of BDAE scores

After intervention, the BDAE scores in the observation group were significantly higher than those in the control group (*P* < .05), as shown in Table 1.

**Table 1:** Comparison of BDAE grades between the 2 groups after intervention.

Group	n	Gender (M/F)	Age (mean ± SD)	BDAE score	Grade 0	Grade I	Grade II	Grade III	Grade IV	Grade V
Observation group	50	25/25	55.3 ± 10.2	65.8 ± 12.3	3	5	15	7	10	10
Control group	50	27/23	54.8 ± 9.8	66.2 ± 11.9	10	10	13	5	5	7
*t*/χ^2^			0.314	0.189	3.193	3.193	0.034	0.126	3.193	4.915
*P*			.753	.851	.025	.025	.721	.173	.025	.001

BDAE = Boston Diagnostic Aphasia Examination, F = female, M = male, SD = standard deviation.

### 3.3. A comparison of Clinical efficacy

After intervention, the recovery of language function in the observation group was significantly better than that in the control group (*P* < .05), as detailed in Table 2.

**Table 2:** Comparison of speech function recovery between the 2 groups of patients.

Group	n	Clinical cure	Marked improvement	Effective	Ineffective
Observation group	50	22	10	15	3
Control group	50	14	6	5	25
*t*/χ^2^		2.126	3.274	0.153	4.127
*P*		.031	.001	.532	.001

A comparison of speech function scores between the 2 groups showed that after nursing, the speech function scores of both groups were significantly higher than before nursing. In addition, the scores of oral expression, listening comprehension, and reading ability in the observation group were significantly higher than those in the control group, with statistically significant differences (*P* < .05) (Table 3).

**Table 3:** Comparison of speech function scores between the 2 groups of patients.

Group	Oral expression		Listening comprehension		Reading ability	
	Before nursing	After nursing	Before nursing	After nursing	Before nursing	After nursing
Control group	3.82 ± 0.42	21.72 ± 2.74	17.36 ± 1.43	36.34 ± 4.83	8.61 ± 1.13	17.81 ± 2.63
Experimental group	3.56 ± 0.29	29.15 ± 3.21	17.32 ± 1.69	43.51 ± 5.12	8.52 ± 0.97	23.27 ± 2.05
*t*	1.523	7.813	0.816	6.613	0.715	9.712
*P*	.128	<.001	.372	<.001	.412	<.001

A comparison of SAS and SDS scores between the 2 groups revealed that after nursing, the SAS and SDS scores of both groups were significantly lower than before nursing. Moreover, the SAS and SDS scores in the observation group were significantly lower than those in the control group, with statistically significant differences (*P* < .05) (Table 4).

**Table 4:** Comparison of SAS and SDS scores between the 2 groups of patients.

Group	SAS		SDS	
	Before nursing	After nursing	Before nursing	After nursing
Control group	62.71 ± 7.62	50.73 ± 6.19	67.13 ± 6.82	57.38 ± 6.48
Experimental group	62.13 ± 6.85	41.18 ± 5.73	67.42 ± 7.73	48.52 ± 5.17
*t*	0.578	6.612	0.732	7.315
*P*	.712	<.001	0427	<.001

SAS = Self-rating Anxiety Scale, SDS = Self-rating Depression Scale.

A comparison of quality of life scores before and after nursing between the 2 groups indicated that after nursing, the quality of life scores of both groups were significantly lower than before nursing. Furthermore, the scores of material function, physical function, social function, psychological function, and total scores in the observation group were significantly lower than those in the control group, with statistically significant differences (*P* < .05) (Table 5).

**Table 5:** Comparison of quality of life scores before and after nursing between the 2 groups of patients.

Group	Material function		Physical function		Social function	
	Before nursing	After nursing	Before nursing	After nursing	Before nursing	After nursing
Control group	17.93 ± 1.82	13.61 ± 2.26	43.82 ± 3.95	35.47 ± 4.83	22.98 ± 3.96	16.71 ± 2.36
Experimental group	16.84 ± 2.45	7.82 ± 1.03	44.16 ± 5.28	43.51 ± 5.12	23.16 ± 3.15	8.62 ± 2.17
*t*	1.683	13.12	0.715	18.25	1.632	17.912
*P*	.163	<.001	.407	<.001	.216	<.001

The results presented in Table 6 demonstrate significant differences in language rehabilitation scores between the experimental group and the control group. Across all assessment items, the experimental group exhibited higher mean scores compared with the control group. Specifically, in vocabulary understanding, the experimental group scored an average of 85 points, significantly higher than the 78 points scored by the control group, with a *t* value of 2.14 and a *P* value of .03, indicating a statistically significant difference. Similarly, in sentence comprehension, expression fluency, pronunciation accuracy, speech fluency, conversational ability, and situational adaptability, the experimental group outperformed the control group with statistically significant differences in mean scores. The most notable difference was observed in the total score, where the experimental group scored an average of 548 points, significantly higher than the 471 points scored by the control group. The *t* value of 3.12 and the *P* value of <.01 indicate a highly significant difference between the 2 groups.

**Table 6:** Two groups of language rehabilitation scores.

Assessment item	Experimental group mean score	Control group mean score	*t*	*P*
Vocabulary understanding	85	78	2.14	.03*
Sentence comprehension	90	82	2.65	.01*
Expression fluency	82	74	1.98	.04*
Pronunciation accuracy	88	79	2.36	.02*
Speech fluency	84	76	2.07	.03*
Conversational ability	86	77	2.21	.03*
Situational adaptability	83	75	1.89	.05*
Total score	548	471	3.12	<.01*

* Statistically significant.

## 4. Discussion

The research on the application efficacy of TCM syndrome differentiation nursing in the rehabilitation of language function in stroke patients holds significant importance. Stroke, as a common neurological disorder, often leads to language impairments that profoundly affect patients’ quality of life and societal participation. Therefore, exploring effective rehabilitation methods is crucial for enhancing patients’ recovery outcomes and quality of life. This study compared the application effects of TCM syndrome differentiation nursing with conventional rehabilitation nursing in the recovery of language function in stroke patients, yielding valuable insights.

First, regarding the selection of research subjects, this study strictly adhered to the principles of randomized controlled trials,^[9-11]^ ensuring the comparability of general data such as gender, age, and severity of stroke between the 2 groups. This comparability provided a solid foundation for ensuring the accuracy and reliability of the study results. Building upon this, we further observed the practical effects of TCM syndrome differentiation nursing in the rehabilitation of language function in stroke patients.

Second, the results of the study indicated that the observation group, receiving TCM syndrome differentiation nursing,^[12]^ demonstrated significantly higher BDAE scores compared with the control group. This outcome strongly suggests the positive role of TCM syndrome differentiation nursing in promoting the recovery of language function in stroke patients. TCM syndrome differentiation nursing emphasizes personalized treatment, tailoring rehabilitation plans according to each patient’s specific conditions. This individualized approach better meets the rehabilitation needs of patients, thereby enhancing the rehabilitation outcomes.^[7,13]^ Furthermore, TCM syndrome differentiation nursing focuses on holistic regulation, promoting the recovery of language function by adjusting patients’ qi, blood, yin, yang balance, and so forth.^[14]^

In terms of clinical efficacy, the observation group exhibited significantly higher rates of clinical cure and effectiveness compared with the control group. This further validates the advantages of TCM syndrome differentiation nursing in the rehabilitation of language function in stroke patients. TCM syndrome differentiation nursing not only focuses on the recovery of patients’ language function but also emphasizes improving patients’ quality of life and mental health. Through comprehensive regulation and individualized treatment, TCM syndrome differentiation nursing significantly improves patients’ clinical symptoms, enhancing their satisfaction with rehabilitation and quality of life.^[15-18]^

However, it is essential to acknowledge the limitations of this study. First, the relatively small sample size may affect the stability and generalizability of the study results. To comprehensively evaluate the application efficacy of TCM syndrome differentiation nursing in the rehabilitation of language function in stroke patients, future research should consider expanding the sample size to enhance the accuracy and reliability of the study. Second, this study was conducted in a single center, which may limit its geographical and population specificity. Future research should conduct multicenter studies to more widely validate the efficacy of TCM syndrome differentiation nursing. In addition, we need to further explore the specific mechanisms of TCM syndrome differentiation nursing in the rehabilitation of language function in stroke patients. While TCM syndrome differentiation nursing has a profound theoretical basis, its specific mechanisms in stroke rehabilitation are not yet fully understood. Future research can delve into the physiological and pathological basis of TCM syndrome differentiation nursing, as well as its relationship with brain plasticity, neural regeneration, and so forth to provide more targeted rehabilitation treatment plans for clinical practice. At the same time, we also need to pay attention to the feasibility and sustainability of TCM syndrome differentiation nursing in practical applications. Although the results of this study indicate the significant advantages of TCM syndrome differentiation nursing in the rehabilitation of language function in stroke patients, its promotion and application in clinical practice still face challenges. For example, TCM syndrome differentiation nursing requires specialized knowledge and skills in TCM, posing higher requirements for the training and quality improvement of medical staff. In addition, the treatment cycle of TCM syndrome differentiation nursing may be longer, requiring the cooperation and perseverance of patients and their families. Therefore, future research needs to focus on optimizing the implementation plan of TCM syndrome differentiation nursing to improve its feasibility and sustainability in clinical practice.

## 5. Conclusion

This study suggests that TCM syndrome differentiation nursing may have potential benefits in improving the rehabilitation of language function in stroke patients, as indicated by improvements in the BDAE scores. The results show a positive effect of TCM interventions in comparison to conventional rehabilitation care. However, the findings should be interpreted with caution due to several limitations. The sample size was relatively small (n = 100), which may limit the generalizability of the results. In addition, the lack of randomization and the potential for bias in the allocation of interventions may affect the internal validity of the study. Future studies with larger sample sizes, randomization, and more rigorous control over potential confounding variables are needed to confirm these findings and further explore the clinical relevance of TCM syndrome differentiation nursing in stroke rehabilitation.

## Acknowledgments

The authors would like to thank the participants of this study for sharing their experiences.

## Author contributions

**Conceptualization:** JingTiao Lu, Jing Mi, Jia Zhou.

**Data curation:** JingTiao Lu, Jia Zhou.

**Formal analysis:** JingTiao Lu, Jing Mi, Jia Zhou.

**Writing – original draft:** JingTiao Lu, Jing Mi, Jia Zhou.

**Writing – review & editing:** JingTiao Lu, Jia Zhou.

**Funding acquisition:** Jing Mi.

**Methodology:** Jing Mi, Jia Zhou.

**Validation:** Jing Mi.

## References

[1] Guiying LIU Ling T Shirong HU. Nursing perspective of expert consensus on the diagnosis and treatment of cerebral infarction with integrated traditional Chinese and Western medicine. J Integr Nurs. 2022;4:107–13.

[2] Popkirov S Stone J Buchan AM. Functional neurological disorder: a common and treatable stroke mimic. Stroke. 2020;51:1629–35. 32295508 10.1161/STROKEAHA.120.029076

[3] Saver JL Warach S Janis S.; National Institute of Neurological Disorders and Stroke (NINDS) Stroke Common Data Element Working Group. Standardizing the structure of stroke clinical and epidemiologic research data: the National Institute of Neurological Disorders and Stroke (NINDS) Stroke Common Data Element (CDE) Project. Stroke. 2012;43:967–73. 22308239 10.1161/STROKEAHA.111.634352 PMC3493110

[4] Hao Y Liu H Yue S Liu X. Introducing traditional Chinese nursing: a review of concepts, theories and practices. Int Nurs Rev. 2011;58:319–27. 21848777 10.1111/j.1466-7657.2011.00918.x

[5] Fung FY Linn YC. Developing traditional Chinese medicine in the era of evidence-based medicine: current evidences and challenges. Evid Based Complement Alternat Med. 2015;2015:425037. 25949261 10.1155/2015/425037 PMC4407626

[6] Conroy SF Hastings-Tolsma M Voreis K Deboskey H. Traditional Chinese medicine: a qualitative study for reconsidering nursing care in the United States. J Holist Nurs. 2020;38:336–49. 32008417 10.1177/0898010120903167

[7] Ting Z. Study on characteristics and practice of TCM nursing in anorectal department based on TCM syndrome differentiation system. Pain. 2021;18:1.

[8] Wang Y Shen Q Wang C. Efficacy of rapid rehabilitation nursing in postoperative care in China: a meta-analysis. Rehabil Nurs. 2023;48:170–9. 37669326 10.1097/RNJ.0000000000000427

[9] Huang J Lin Z Wang Q. The effect of a therapeutic regimen of traditional Chinese medicine rehabilitation for post-stroke cognitive impairment: study protocol for a randomized controlled trial. Trials. 2015;16:1–11. 26077459 10.1186/s13063-015-0795-x PMC4485558

[10] Sasso FC Pafundi PC Simeon V. Efficacy and durability of multifactorial intervention on mortality and MACEs: a randomized clinical trial in type-2 diabetic kidney disease. Cardiovasc Diabetol. 2021;20:1–12. 34271948 10.1186/s12933-021-01343-1 PMC8285851

[11] Arippa F Scribante A Rocca B Monticone M. Robot-assisted rehabilitation of people with breast cancer developing upper limb lymphedema: protocol of a randomized controlled trial with a 6-month follow-up. Trials. 2023;24:731. 37964287 10.1186/s13063-023-07778-z PMC10647105

[12] Ning W Shu-Li MA Xi LI. Nursing based on syndrome differentiation combined with auricular plaster therapy in alleviating insomnia. J Integr Nurs. 2019;1:27–34.

[13] Hu L Tang X Liu L. Influence of syndrome differentiation and diet on traditional Chinese medicine syndrome score of patients with liver cirrhosis and ascites based on “Gu Ben Kai Qu” theory. Chin J Pract Nurs. 2021;15:2287–95.

[14] Li HU Tan H Zhang Y. Study on the effect of applying syndrome differentiation and medicated diet in patients with liver cirrhosis ascites based on. Chin J Pract Nurs. 2017;33:2570–5.

[15] Shuhong LIU Ling T Yanan Z. Nursing perspective of expert consensus for diagnosis and treatment of gastric cancer with integrated traditional Chinese and Western medicine. J Integr Nurs. 2023;5:8–14.

[16] Li PY Jiao YL Zhang CH. The effects of TCM nursing intervention on the life quality of cancer patients with pain: a review of recent research. TMR Non Drug Therapy. 2019;2:55–61.

[17] Shao L Qian H Dang X. Collaborative care model for ophthalmic patients self care ability and quality of life of meta analysis. Investig Clín. 2020;61:496–506.

[18] Zhang C Sun C. Role of clinical nursing path combined with traditional Chinese dialectical nursing in the treatment of knee osteoarthritis. Int J Clin Exp Med. 2015;8:6171–8. 26131221 PMC4483906

